# Netrin-1 directs dendritic growth and connectivity of vertebrate central neurons *in vivo*

**DOI:** 10.1186/s13064-015-0041-y

**Published:** 2015-06-10

**Authors:** Anastasia N. Nagel, Sonya Marshak, Colleen Manitt, Rommel A. Santos, Marc A. Piercy, Sarah D. Mortero, Nicole J. Shirkey-Son, Susana Cohen-Cory

**Affiliations:** Department of Neurobiology and Behavior, University of California, 2205 McGaugh Hall, Irvine, CA 92697-4550 USA; Present address: Phamatech, Inc., 15175 Innovation Dr., San Diego, CA 92128 USA; Present address: Department of Biology, St. Olaf College, 1520 St. Olaf Avenue, Northfield, MN 55057 USA

**Keywords:** *In vivo* imaging, DCC, UNC-5, Dendritogenesis, *Xenopus laevis*, Optic tectum

## Abstract

**Background:**

Netrins are a family of extracellular proteins that function as chemotropic guidance cues for migrating cells and axons during neural development. In the visual system, netrin-1 has been shown to play a key role in retinal ganglion cell (RGC) axon growth and branching at the target, where presynaptic RGC axons form partnerships with the dendrites of tectal neurons. However, the signals that guide the connections between RGC axons and their postsynaptic partners are yet unknown. Here, we explored dynamic cellular mechanisms by which netrin-1 influences visual circuit formation, particularly those that impact postsynaptic neuronal morphology and connectivity during retinotectal wiring.

**Results:**

Time-lapse *in vivo* imaging of individual *Xenopus laevis* optic tectal neurons co-expressing tdTomato and PSD95-GFP revealed rapid remodeling and reorganization of dendritic arbors following acute manipulations in netrin-1 levels. Effects of altered netrin signaling on developing dendritic arbors of tectal neurons were distinct from its effects on presynaptic RGC axons. Within 4 h of treatment, tectal injection of recombinant netrin-1 or sequestration of endogenous netrin with an UNC-5 receptor ectodomain induced significant changes in the directionality and orientation of dendrite growth and in the maintenance of already established dendrites, demonstrating that relative levels of netrin are important for these functions. In contrast, altering DCC-mediated netrin signaling with function-blocking antibodies induced postsynaptic specialization remodeling and changed growth directionality of already established dendrites. Reducing netrin signaling also decreased avoidance behavior in a visually guided task, suggesting that netrin is essential for emergent visual system function.

**Conclusions:**

These *in vivo* findings together with the patterns of expression of netrin and its receptors reveal an important role for netrin in the early growth and guidance of vertebrate central neuron dendritic arbors. Collectively, our studies indicate that netrin shapes both pre- and postsynaptic arbor morphology directly and in multiple ways at stages critical for functional visual system development.

## Background

Netrins are members of an evolutionarily conserved family of laminin-related proteins that play important roles during nervous system development [1]. Netrins can be attractive or repulsive depending upon the receptors expressed by responding cells [2, 3]. In vertebrates, the deleted in colorectal cancer (DCC) family of receptors generally mediates chemoattractant responses to netrin-1 but can also contribute to chemorepellent signaling when acting together with uncoordinated-5 (UNC-5) [4, 5]. UNC-5 receptors can mediate chemorepulsion from netrin-1 in both a DCC-dependent and DCC-independent manner [6–8].

The majority of studies on the role of netrin-1 as a guidance molecule have focused on its effects on axon growth and branching. In the vertebrate visual system, netrin guides retinal ganglion cell (RGC) axons along the visual pathway [9]. *In vitro* and *in vivo* studies in *Xenopus* embryos further show that RGC axons exhibit differential responses to netrin-1 that depend on their location along the pathway and on their maturational stage [10–12]. At younger developmental stages, when RGC axons first reach their target, netrin-1 halts growth cone advancement and induces back branching [12]. In contrast, netrin affects mature RGC axons that actively arborize within the target by promoting axonal maturation in a DCC-dependent manner by increasing presynaptic differentiation and dynamic branching [11]. Studies in *Drosophila melanogaster* and *Caenorhabditis elegans* show that in addition to influencing growing axons, netrin can also affect dendritic outgrowth and targeting [13–15].

Here, we investigated potential *in vivo* roles of netrin-1 during the differentiation of postsynaptic neuron dendritic arbors in the vertebrate brain. *In situ* hybridization and immunohistochemistry revealed a restricted pattern of netrin-1 mRNA expression and the localization of DCC and UNC-5 receptors in subpopulations of neurons in the *Xenopus* optic tectum, suggesting that tectal neurons, comparable to RGC axons, can also respond directly to endogenous netrin-1. *In vivo* imaging of individual neurons co-expressing tdTomato and PSD95-GFP showed that acute changes in netrin-1 levels induce rapid dynamic reorganization of tectal neuron dendrites and a change in the directionality of dendrite growth by increasing new branch addition and by destabilizing existing dendrites. Similar to the effects of netrin-1, blocking DCC-mediated netrin-1 signaling altered the formation and maintenance of postsynaptic specializations but changed the directionality of dendrite growth by altering the orientation of stable dendrites only. To correlate effects on neuron morphology with changes in visual function, we examined the behavior of tadpoles in a visual avoidance task. Together, these experiments indicate that netrin-1 signaling is required for the stability and proper orientation of developing tectal neuron dendrites and for their proper connectivity and function. Consequently, by differentially influencing both pre- and postsynaptic cells, netrin-1 can shape neuronal connectivity during early wiring events that establish the visual system.

## Results

### Expression of netrin-1 and its receptors in the tectum during visual circuit development

**Fig. 1:**
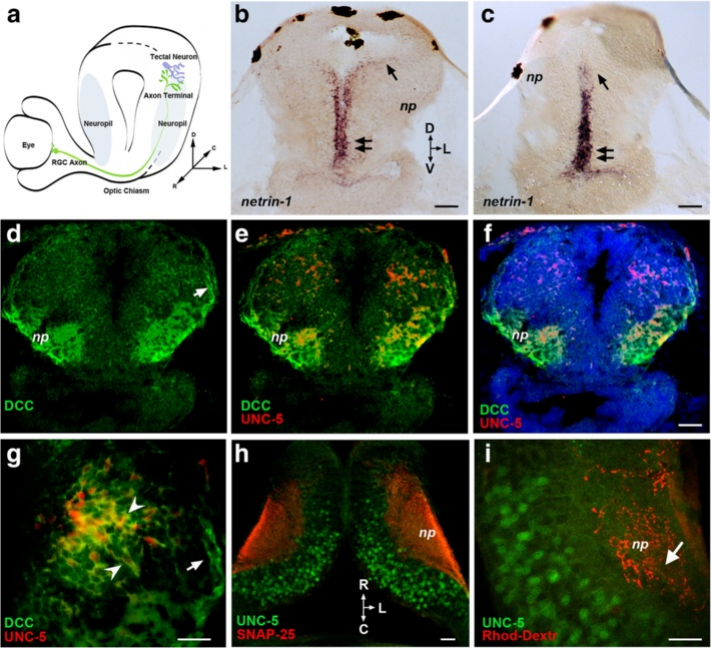
Expression of netrin-1 and of its receptors DCC and UNC-5 in stage 45 *Xenopus* optic tectum. **a** Schematic of coronal section of *Xenopus* retinotectal circuit. RGC axons (*green*) travel from the contralateral eye to connect with tectal neurons in the neuropil (*blue*). **b, c** *In situ* hybridization with *Xenopus*-specific antisense netrin-1 probes. Coronal sections of the midbrain at the level of the optic tectum show ventral-high (*double arrows*) to dorsal-low (*arrow*) netrin-1 mRNA expression along the ventricle wall. **d–g** Coronal and **h, i** horizontal sections show DCC and UNC-5 expression. **d–g** Co-immunostaining illustrates the differential distribution of UNC-5 (*red*) and DCC (*green*) immunoreactivity. **d** DCC immunoreactivity (*green*) is localized to the cell bodies in the dorsal tectum and proximal dendrites and to incoming axons near the dorsal neuropil (*arrow*). The tectal neuropil (*np*) is also positive for DCC. The low- (**e, f**) and high- (**g**) magnification coronal images show UNC-5 (*red*) and DCC (*green*) co-localization, with UNC-5 being localized to a subset of cells that also expresses DCC (**g**, *arrowheads*). **f** Counterstaining with DAPI (*blue*) serves to distinguish nuclear staining from cytoplasmic UNC-5 (*red*) and DCC (*green*) expression in tectal cells. **h** UNC-5 immunoreactivity (*green*) is localized to a subset of cell bodies in the dorsal area of the tectum and area adjacent to the tectal neuropil identified by immunostaining with antibodies to the presynaptic protein SNAP-25 (*red*). **i** Anterograde labeling with rhodamine dextran shows that RGC axons (*red*) terminate in the areas of the tectal neuropil (*arrow*) where UNC-5 immunopositive neurons localize (*green*). *D* dorsal, *V* ventral, *C* caudal, *R* rostral, *L* lateral, *np* neuropil. Scale bars: 50 μm in **b–f**, 20 μm in **g**, 20 μm in **h–i**

**Fig. 2:**
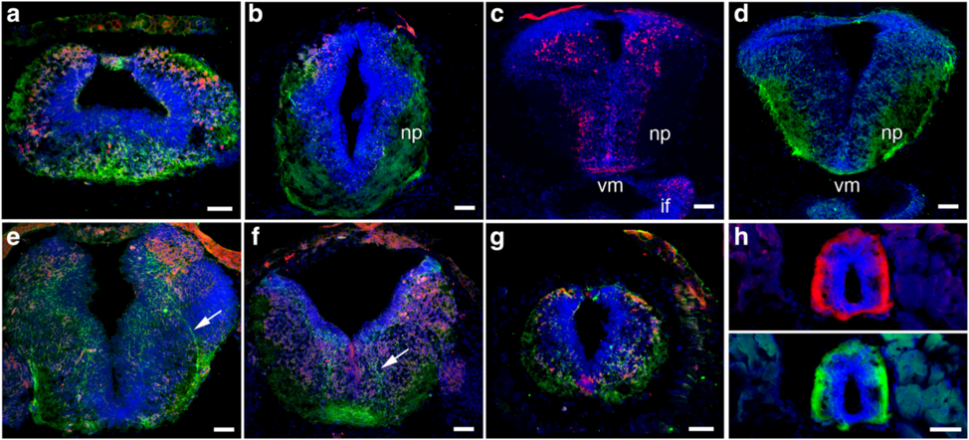
Specific patterns of DCC and UNC-5 expression in the *X. laevis* central nervous system. Immunostaining with antibodies to UNC-5 (*red*) and DCC (*green*) revealed specific patterns of expression of the netrin-1 receptors in stage 45 tadpoles. **a–g** UNC-5 (*red*) and DCC (*green*) immunoreactivity in the forebrain (**a**), pre-tectum (**b**), caudal tectum (**e**), hindbrain (**f**), and rostral spinal cord (**g**) demonstrate a specific pattern of expression for each of these receptors within subpopulations of neurons in the central nervous system. **c** UNC-5 immunostaining (*red*) localizes to subpopulations of neurons in the dorsal tectum, lateral-ventral midbrain, ventral midline (*vm*), and infundibulum (*if*). **d** DCC immunoreactivity (*green*) is localized in dorsal tectal neuron cell bodies and processes in the tectum and ventral midline, as well as in the tectal neuropil (*np*). **e, f** Note the specificity of immunostaining and co-localization of UNC-5 and DCC expression in subpopulations of cells in the caudal tectum (**e**) and hindbrain (**f**) and the localization of DCC receptors to discrete fiber tracts (*arrows*). **g, h** UNC-5 (*red*) and DCC (*green*) immunoreactivity in the rostral (**g**) and caudal (**h**) spinal cord is localized to fiber tracts and ventral midline in agreement with published observations in *Xenopus* and other species (for review, see [42, 5, 43–45]). DCC immunoreactivity in the spinal cord is similar when staining with antibodies directed against the extracellular (**g**) or intracellular (**h**, bottom) domains of DCC. Counterstaining with DAPI (*blue*) serves to distinguish nuclear staining from UNC-5 (*red*) and DCC (*green*) expression in cell bodies and fiber tracts. Scale bars: 50 μm

In the developing *Xenopus* visual system, RGC axons at their target express DCC and differentially respond to netrin-1 depending on their maturational state by halting growth cone advancement within the target [12] or by rapidly increasing the number of green fluorescent protein (GFP)-tagged presynaptic specializations and subsequently increasing branch number [11]. To further characterize the roles of netrin-1 during visual circuit development, we examined the expression of netrin-1 and its receptors DCC and UNC-5 in the optic tectum at the time when tectal neurons differentiate and form connections with branching RGC axons (Fig. 1a). Quantitative reverse transcription polymerase chain reaction (RT-PCR) showed DCC, UNC-5, and netrin-1 mRNA expression in the midbrain of stage 41 to 45 tadpoles (not shown). *In situ* hybridization studies revealed that netrin-1 mRNA is expressed in the midbrain of stage 45 tadpoles predominantly near the ventricle wall, in a ventral-high to dorsal-low gradient (Fig. 1b, c). Immunostaining with antibodies to UNC-5 and to DCC demonstrated areas of overlapping expression for these two netrin-1 receptors within the midbrain at this same stage (Fig. 1d–g). In the optic tectum, UNC-5 immunoreactivity was restricted primarily to cell bodies and proximal processes in the dorso-caudal midbrain (Figs. 1e–i, 2c) and was absent from the tectal neuropil where presynaptic retinal ganglion cell (RGC) axons terminate (Fig. 1h, i). Immunostaining with an antibody against the extracellular domain of DCC revealed that DCC was localized throughout the tectal neuropil (Fig. 1d–g, Fig. 2d), consistent with findings using antibodies that recognize the intracellular domain of DCC [11]. Moreover, DCC immunoreactivity was found around tectal cell bodies and in neuronal processes that extended to the tectal neuropil where primary dendrites begin to branch. Defined patterns of UNC-5 and DCC expression were also found in the forebrain, pre-tectum, caudal tectum, hindbrain, and spinal cord (Fig. 2). Consequently, the patterns of netrin-1 mRNA (Fig. 1b, c) and protein expression [11] and the localization of DCC and UNC-5 receptors within the optic tectum suggest that tectal neurons can respond to netrin-1 directly.

### Acute manipulations in netrin levels or DCC signaling

**Fig. 3:**
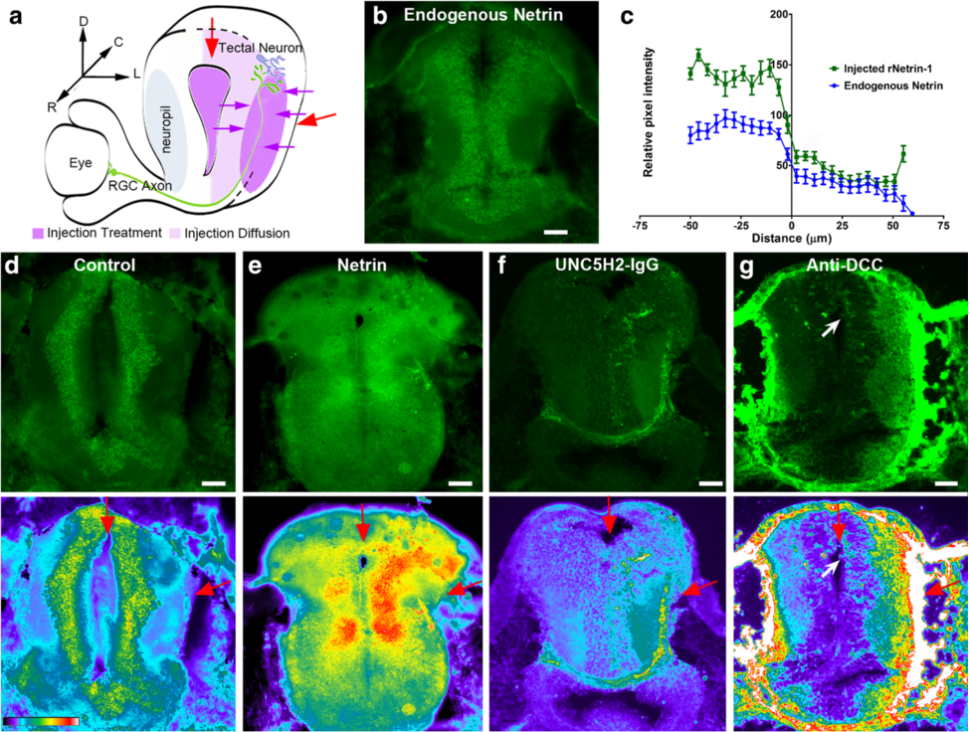
Protein diffusion after treatment. **a** Schematic of coronal view of stage 45 *Xenopus* retinotectal circuit depicting injection sites (*red arrows*) and spread of injected proteins (*violet color*). **b** Coronal section at the level of the optic tectum immunostained with antibodies to netrin-1. Note endogenous netrin immunoreactivity in cell body layer and neuropil. **c–g** Sections at the level of the optic tectum of tadpoles injected with vehicle, recombinant netrin-1, UNC5H2-Ig, or anti-DCC were immunostained to examine the spread of the injected proteins after treatment. **c** Quantitative analysis of fluorescence intensity in sections of uninjected tadpoles (*Endogenous Netrin*) or tadpoles injected with recombinant netrin (*Injected rNetrin-1*). The relative levels of netrin within the cell body layer and the neuropil are illustrated by the average pixel intensity values along the medial-to-lateral axis of the tectum. The zero value in the *X*-axis corresponds to the cell body layer-neuropil boundary; negative *X*-coordinates represent distance from the boundary to the ventricle while positive *X*-coordinates represent distance from the boundary to the lateral-most neuropil. *n* = 10 brain sections per group, from four tadpole brains per group, with three 20-pixel-wide line scans quantified per section. Error bars represent the standard error of the mean. **d–g** Sample coronal sections of tadpoles injected with vehicle (**d**), recombinant netrin-1 (**e**), UNC5H2-Ig (**f**), or anti-DCC (**g**) immunostained with chick antibodies to netrin-1 and Alexa 488 secondary antibodies to chick IgG (top; **d, e**) or stained with Alexa 488 secondary antibodies to human IgG (top; **f**) or mouse IgG (top; **g**). The pseudo-color images in **d–g** (bottom) show the relative intensity of the Alexa fluor 488 fluorescence. Pixel intensity values ranged from 0 (black) to 255 (white) as illustrated by the color-scale bar (**d**, bottom). Note the increased immunofluorescence in the cell body layer and neuropil of netrin-1-treated tadpoles (**e**) when compared to vehicle-injected controls (**d**) and with endogenous netrin-1 expression (**b**). In **f** and **g**, the relatively higher fluorescence intensity in the hemisphere that received the injection (*red arrows*) and the diffusion patterns of the proteins are more evident in the pseudo-color images. In **g**, *white arrows* point to fluorescently labeled cells in the injected tectal hemisphere. Scale bars in **b**, **d–g**: 50 μm

To explore dynamic mechanisms by which netrin-1 influences postsynaptic neuronal morphology and connectivity in the retinotectal system, we altered endogenous netrin-1 levels or DCC signaling in the stage 45 tadpole optic tectum by microinjecting recombinant netrin-1, an UNC-5 receptor ectodomain that sequesters netrin (UNC5H2-Ig), or function-blocking antibodies to DCC. We examined protein distribution immediately after injection to determine rates of diffusion from the injection site (Fig. 3) as a means to evaluate the effectiveness of the acute treatments. Immunostaining with specific antibodies to netrin revealed that netrin-1 injection into the ventricle and lateral side of the optic tectum resulted in higher immunoreactivity in the neuropil near the injection site and an even distribution of the exogenous protein within the cell body layer above the endogenous netrin expression (Fig. 3b–e). Quantitative analysis of the immunofluorescent signal further demonstrated that the treatment was effective in increasing tectal netrin levels (Fig. 3c). Staining with a fluorescent antihuman IgG antibody allowed visualization of the injected UNC5H2 Fc chimeric protein and showed graded distribution of UNC5H2-Ig in the tectal hemisphere that received treatment (Fig. 3f), which could alter the localized spatial distribution of endogenous netrin-1. Similarly, immunostaining with fluorescent anti-mouse IgG to visualize the injected function-blocking antibody to DCC demonstrated that anti-DCC effectively diffused within the neuropil (Fig. 3g) and had the ability to bind the endogenous receptor and prevent signaling.

### Netrin differentially affects retinal ganglion cell axons and tectal neuron dendrites

**Fig. 4:**
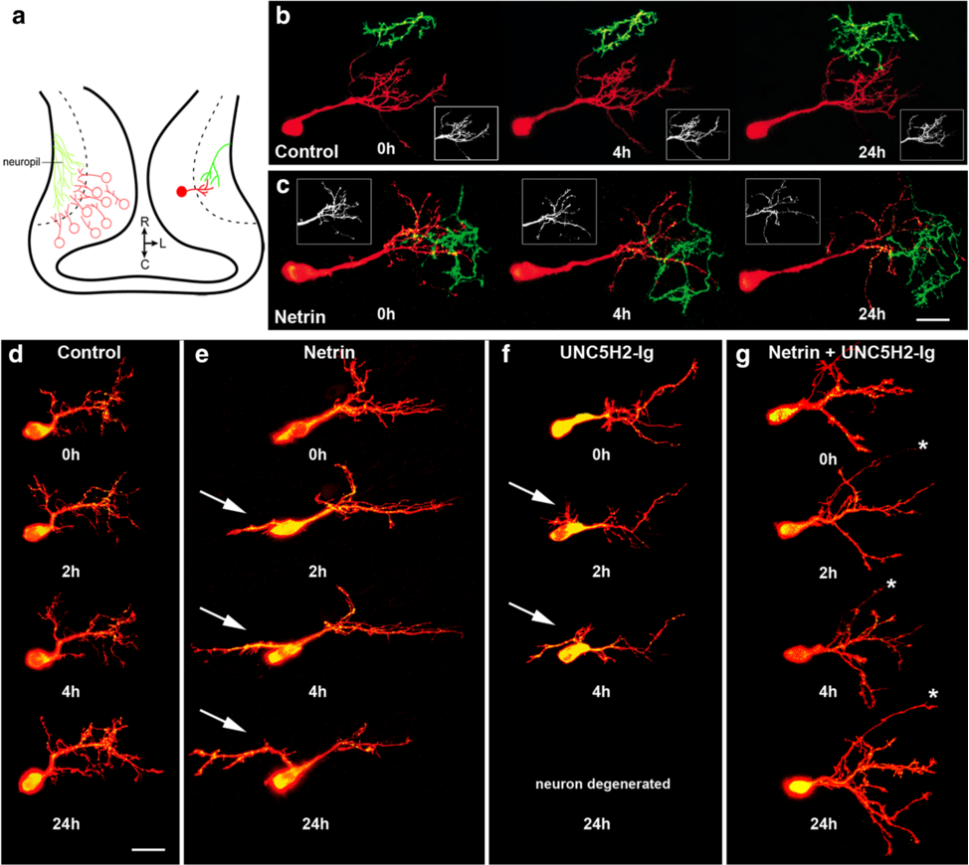
Rapid remodeling of dendritic arbors upon acute manipulations in netrin signaling. **a** Schematic diagram of a stage 45 *Xenopus* tectal midbrain (horizontal view). Tectal neurons (*red*) make dendritic connections with contralateral RGC axons (*green*) within the tectal neuropil. **b, c** Sample RGC axons and tectal neurons, visualized by expression of GFP and tdTomato, respectively, in control (**b**) and netrin-treated (**c**) tadpoles. Note change in tectal neuron dendritic architecture evident at 4 and 24 h after netrin-1 treatment (*inserts*). **d–g** Confocal projections of representative tectal neurons co-expressing tdTomato (*red*) and PSD95-GFP (*green*) in tadpoles injected with control vehicle solution (**d**), Netrin (**e**), UNC5H2-Ig (**f**), or Netrin + UNC5H2-Ig (**g**). Note the emergence of an alternative primary dendrite (*arrow*) growing towards the midline in neurons exposed to netrin-1 or UNC5H2-Ig. Tadpoles treated with netrin + UNC5H2-Ig appeared identical to controls. Axons of tectal neurons are labeled by the *asterisks*. Scale bars: 20 μm

To examine if netrin-1 shapes postsynaptic neuronal connectivity in addition to influencing RGCs, we imaged pairs of fluorescently labeled pre- and postsynaptic arbors branching in the optic tectum of stage 45 tadpoles. The simultaneous, dynamic behavior of individual tectal neurons expressing tdTomato and of RGC axons expressing GFP was followed *in vivo* by confocal microscopy (Fig. 4a). In control tadpoles, both presynaptic and postsynaptic arbors gradually grew towards one another within the tectal neuropil (Fig. 4b). Upon acute injection of recombinant netrin-1, however, tectal neurons showed rapid reorganization of their dendritic arbor (Fig. 4c) while RGC axons continued to grow forward and elaborate. Dendrites of tectal neurons appeared to alter their branch directionality away from the neuropil and from branching RGC axons (Fig. 4c, insets). As tectal neurons responded to recombinant netrin-1 by remodeling their dendritic arbors, RGC axons increased their number of branches significantly more than controls 24 h after netrin-1 treatment (control 170.5 ± 13.79 % *n* = 4, netrin 247.8 ± 15.93 % *n* = 4, *p* = 0.0105; not shown graphically) in agreement with previous findings [11].

### Effects of netrin-1 on the morphological development of developing tectal neurons

**Fig. 5:**
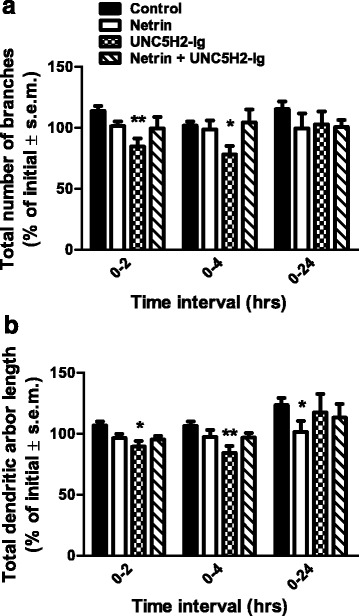
Altering endogenous netrin levels decreases dendrite branch number and total dendritic arbor length. Effects of tectal microinjection of netrin, UNC5H2-Ig, or netrin + UNC5H2-Ig on total dendrite branch number (**a**) and length (**b**). Netrin-1 and UNC5H2-Ig altered tectal neuron morphology with a different time scale. Note that exogenous netrin-1 treatment decreased dendrite arbor length at 24 h, while the UNC5H2-Ig treatment that sequesters endogenous netrin induced a transient but significant decrease in branch number at the 0- to 2- and 0- to 4-h imaging intervals when compared to all other treatments. Co-treatment with netrin + UNC5H2-Ig did not influence branch number or length. Values are expressed as percent change from the initial 0-h imaging session. Two-way ANOVA with Bonferroni multiple comparison test; *p < 0.05, **p < 0.01. Error bars indicate SEM

To further characterize the differential response of tectal neurons to netrin-1, we imaged individual neurons co-expressing tdTomato and PSD95-GFP before (time 0), 2, 4, and 24 h after netrin-1 treatment. Control neurons extended their dendritic arbor without altering their basic architecture (Fig. 4b, d). In contrast, neurons in tadpoles treated with netrin-1 rapidly reorganized their dendritic arbors (Fig. 4c, e). Quantitative analysis of dendrite branching showed that treatment with exogenous netrin-1 did not significantly influence total branch number or dendritic arbor length of tectal neurons (Fig. 5a, b). One possibility that could account for the effects of acute netrin-1 treatment on dendritic arbor shape is that activation of netrin signaling increased the exploratory activity of dendritic processes which leads to a dynamic reorganization of the arbor without affecting overall branch growth. To further explore the effects of netrin, we decreased endogenous netrin levels in the tectum by injecting UNC-5 receptor bodies (UNC5H2-Ig) as a means to sequester bioavailable netrin-1 [16]. Injection of UNC5H2-Ig into the midbrain ventricle and the lateral side of the tectum also caused rapid reorganization and reorientation of tectal neuron dendritic arbors (Fig. 4f). Moreover, UNC5H2-Ig treatment significantly decreased total branch number and dendrite arbor length by 2 h, an effect that was maintained 4 h after treatment (Fig. 5a, b). Consequently, tectal neurons responded to decreased tectal netrin levels more robustly but similarly to exogenous netrin-1, suggesting that the destabilization and reorientation of dendrites may be attributed to the disruption of differential endogenous netrin expression or signaling. To further test for specificity of effects, we co-injected tadpoles with a mix of netrin-1 and UNC5H2-Ig at a ratio in which recombinant netrin-1 would neutralize the UNC-5 ectodomain dimer (1.7:2 mol:mol solution). In contrast to netrin-1 treatment alone or UNC5H2-Ig treatment alone, neurons in tadpoles co-treated with netrin and UNC5H2-Ig had morphologies and total branch number and length indistinguishable from controls (Fig. 4g; Fig. 5a, b). Therefore, our studies indicate that while responses to exogenous netrin-1 and to sequestration of endogenous netrin with the UNC-5 ectodomain are similar, they are specific to each treatment.

### DCC-mediated signaling influences dendritic growth and directionality without altering total branch number or length

**Fig. 6:**
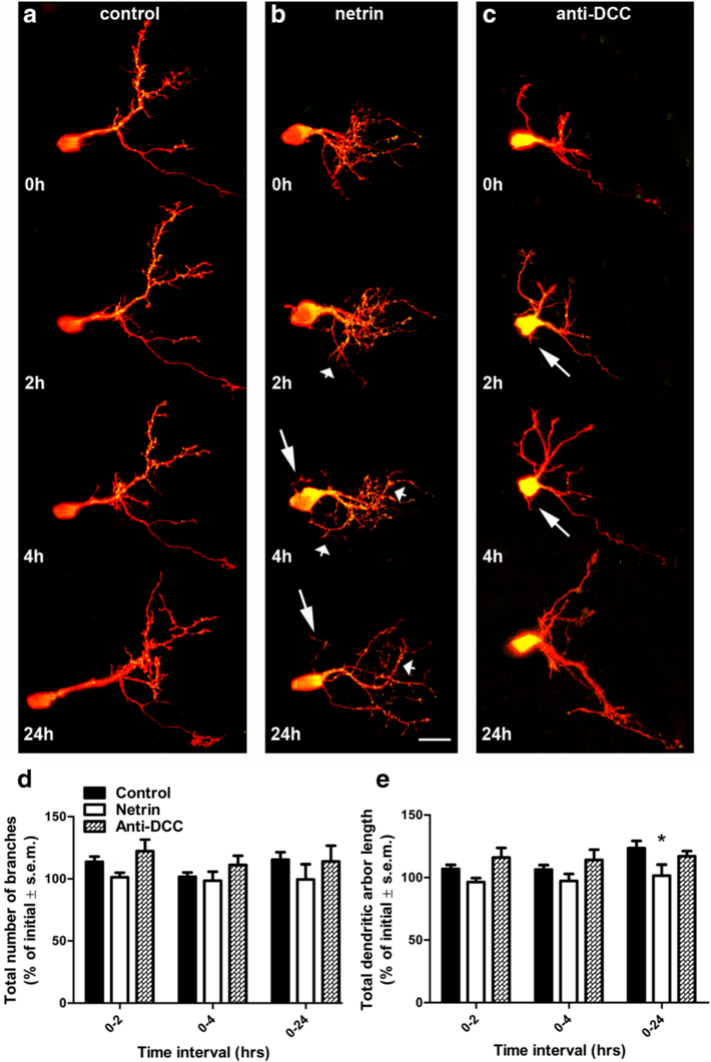
Blocking DCC signaling induces changes in dendritic arbor shape without altering total branch number or length. **a–c** Confocal projections of representative tectal neurons co-expressing tdTomato (*red*) and PSD95-GFP (*green*) in tadpoles injected with control vehicle solution (**a**), netrin-1 (**b**), or function-blocking antibodies to DCC (**c**). While control neurons branch, elaborate, and add PSD95-GFP puncta (**a**), neurons in tadpoles treated with netrin-1 undergo dynamic remodeling of existing branches (**b**). *Short arrows* point to dendrites with altered directions of growth. Neurons in tadpoles treated with anti-DCC (**c**) also appear to change dendritic arbor direction and form small basal projections at 2 and 4 h post-injection (*long arrows*). Scale bars: 20 μm. **d, e** Comparison of effects of netrin and anti-DCC on total branch number (**d**) and dendritic arbor length (**e**). Note that only netrin-1 treatment decreased arbor length at 24 h (**e**), but neither netrin nor anti-DCC affects the total number of branches (**d**). Two-way ANOVA with Bonferroni multiple comparison test; *p < 0.05. Error bars indicate SEM

In *Xenopus*, RGC axons respond to altered DCC receptor signaling at their target by halting their presynaptic differentiation and growth [11, 12]. To determine whether the effects of altered netrin levels on tectal neurons are also mediated through its receptor DCC, we examined dynamic changes in arbor morphology of tectal neurons following injection of function-blocking antibodies to DCC. Neurons in tadpoles treated with anti-DCC rapidly remodeled their dendritic arbors and changed their morphology when compared to controls (Fig. 6a, c) similarly but less robustly than the effects of netrin-1 (Figs. 4e, 6b). As observed for neurons in tadpoles treated with netrin-1 or with UNC5H2-Ig, anti-DCC induced the formation of ectopic basal projections in tectal neurons 2 and 4 h after treatment (Fig. 6b, c (arrows), see also Fig. 4). However, in contrast to treatment with the UNC-5 ectodomain, anti-DCC treatment did not alter total dendrite branch number or total arbor length at any imaging interval (Fig. 6d, e).

### Altering endogenous netrin signaling induces rapid remodeling of dendritic arbors

**Fig. 7:**
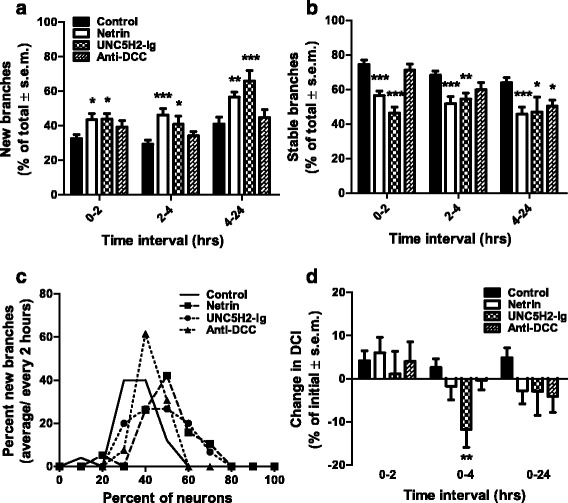
Acute manipulations in endogenous netrin levels induce rapid changes in dendrite remodeling. **a, b** Effects of netrin-1, UNC5H2-Ig, or anti-DCC treatments on new branch addition (**a**) and branch stabilization (**b**). Note that while netrin-1 and UNC5H2-Ig increased branch addition and decreased branch stabilization throughout the 24-h imaging period, the anti-DCC treatment influenced the stability of branches at the 4- to 24-h interval only. **c** Relative proportion of neurons with different branch addition rates. A significant shift in the distribution of neurons that responded with increased branch addition rates was observed after netrin-1 and UNC5H2-Ig treatments. Values are expressed as percent change from total branches. **d** Relative change in DCI values is shown for each group at all imaging intervals. Note that neurons in UNC5H2-Ig-treated tadpoles significantly decreased their complexity by 4 h compared to controls. Two-way ANOVA with Bonferroni multiple comparison test; *p < 0.05, **p < 0.01, ***p < 0.001. Error bars indicate SEM

Neurons in tadpoles treated with UNC5H2-Ig responded to decreasing netrin-1 levels by altering total branch number and length early after treatment. However, treatment with netrin-1 or anti-DCC caused remodeling of dendritic arbors without influencing total branch number or length. To further characterize the differences in tectal neuron responses to altered netrin-1 levels and DCC signaling, we analyzed branch dynamics of tectal neurons imaged over the 24-h period. Detailed quantitative analysis demonstrated that tectal neurons responded to netrin-1 and to UNC5H2-Ig through similar dynamic reorganization of their dendritic arbors. Neurons in netrin-1- and in UNC5H2-Ig-treated tadpoles increased new branch addition and decreased branch stabilization (Fig. 7a, b). Significantly more branches were added following netrin-1 or UNC5H2-Ig treatments relative to controls at all time intervals (Fig. 7a) while the stability of existing branches was also decreased (Fig. 7b). A similar shift in the distribution of neurons that responded to netrin-1 or UNC5H2-Ig with increased branch addition rates further demonstrates that neurons responded similarly to these treatments independent of their initial morphology and branch number (Fig. 7c). The rapid changes in branch addition and stability following treatment with netrin-1 alone or with UNC5H2-Ig alone therefore suggest that threshold levels of netrin protein or receptor-mediated signaling contribute to these remodeling effects. In contrast to netrin-1 and to UNC5H2-Ig, the anti-DCC treatment only induced a small but significant decrease in the stability of branches by 24 h (Fig. 7b). As for other measures, tadpoles treated with netrin and UNC5H2-Ig in combination had branch addition and branch stabilization rates similar to controls at all imaging intervals (addition 0–2 h, control 32.58 ± 2.25 %, netrin + UNC5H2-Ig 29.99 ± 3.44 %; stabilization 0–2 h, control 74.57 ± 2.45 %, netrin + UNC5H2-Ig 71.20 ± 5.72, *p* > 0.05 two-way ANOVA, not shown graphically), supporting the specificity of the individual treatments. Together, these results demonstrate that alterations in tectal netrin levels significantly influenced the dynamic remodeling of dendritic arbors while dendrites continued to remodel at a similar rate but failed to stabilize following blockade of DCC signaling.

To further evaluate the morphological changes in neurons elicited by altered netrin levels and signaling, we calculated dendritic complexity index (DCI) [17], a measure of the relative proportion of primary, secondary, and higher order branches. The complexity of neurons in UNC5H2-Ig-treated tadpoles was significantly lower than controls 4 h after treatment, as shown by the relative change in DCI values between 0 and 4 h after treatment (control 2.589 ± 1.978 % vs. UNC5H2-Ig −11.760 ± 4.145 %, *p* < 0.01; two-way ANOVA with Bonferroni multiple comparison, Fig. 7d). We further examined whether the decrease in dendritic arbor complexity was due to changes in the addition of lower order branches or to elimination of higher order branches by quantifying the proportion of primary, secondary, tertiary, and higher order branches for each neuron. Correspondingly, the number of tertiary branches in neurons in UNC5H2-Ig-treated tadpoles was significantly lower than in controls 4 h after treatment (absolute numbers: control 5.962 ± 0.5363 vs. UNC5H2-Ig 2.933 ± 0.6053, *p* < 0.001; two-way ANOVA with Bonferroni multiple comparison, not shown graphically), and in proportion, tertiary branches were also lower than in controls (tertiary branches: control 35.059 ± 2.769 % vs. UNC5H2-Ig 22.995 ± 3.675 %, *p* < 0.05; primary branches: control 11.258 ± 1.355 % vs. UNC5H2-Ig 23.076 ± 6.477 % *p* < 0.05; two-way ANOVA with Bonferroni multiple comparison, not shown graphically). Neurons from tadpoles treated with anti-DCC also had a significantly lower number and proportion of tertiary branches relative to controls at 24 h (tertiary branches: control 6.389 ± 0.805, anti-DCC 3.000 ± 0.768, *p* < 0.001; control 34.092 ± 2.78 %, anti-DCC 20.860 ± 4.00 %, *p* < 0.01; two-way ANOVA with Bonferroni multiple comparison, not shown graphically). The change in number and proportion of tertiary branches in neurons in anti-DCC-treated tadpoles is consistent with the time when stable branches were also significantly decreased, although the DCI values did not differ significantly in this group from that of controls. Consequently, the changes in the dendritic arbor complexity and pruning of higher order branches reflect the active remodeling of the dendritic arbors in response to decreased netrin levels or DCC signaling.

### Netrin influences the dynamics and maintenance of postsynaptic specializations

**Fig. 8:**
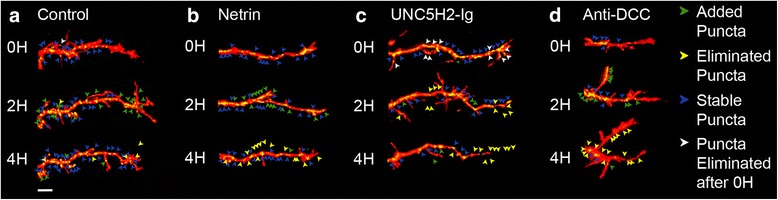
Altered netrin-1 levels and DCC signaling impact postsynaptic cluster remodeling. **a–d** Confocal projections of single branches from representative tectal neurons co-expressing tdTomato (*red*) and PSD95-GFP (*green*) from control (**a**), netrin (**b**), UNC5H2-Ig (**c**), or Anti-DCC (**d**) groups before and after treatment. Dynamic remodeling of postsynaptic specializations is illustrated by the addition (*green arrowheads*) and elimination (*yellow arrowheads*) of PSD95-GFP clusters. *Blue arrowheads* denote puncta that remained stable from one observation interval to the next; *white arrowheads* denote puncta that were present at the initial observation time point but were eliminated (*yellow*) at 2 h. Scale bar: 20 μm

**Fig. 9:**
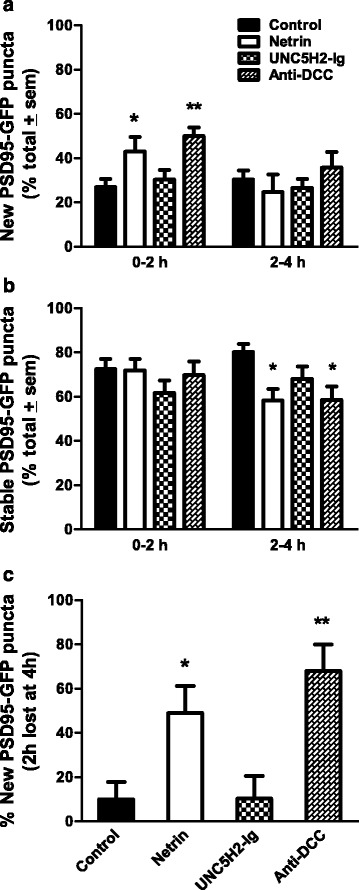
Postsynaptic cluster addition and stabilization are modulated by alterations in netrin signaling. **a, b** Effects of netrin-1, UNC5H2-Ig, or anti-DCC treatments on postsynaptic cluster remodeling were quantified as the proportion of PSD95-GFP puncta that were added (**a**) and remained stable (**b**) within the 0–2 and 2–4 observation intervals. Note that significantly more PSD95-GFP puncta were between 0 and 2 h (**a**), while fewer were stable between 2 and 4 h (**b**) following netrin-1 or anti-DCC treatment when compared to controls. **c** To determine the relative stability of newly added postsynaptic clusters, we quantified relative proportion of PSD95-GFP puncta added over the 0- to 2-h interval that were lost in the subsequent 2- to 4-h interval for a subset of randomly selected neurons for each group (*n* = 4). PSD95-GFP puncta added from 0 to 2 h were significantly less stable in the netrin-1- or anti-DCC-treated neurons. Statistical significance was by one-way ANOVA and with unpaired *t*-tests. Significance when compared to control is *p < 0.05, **p < 0.01. Error bars indicate SEM

*In vivo* imaging studies in *Xenopus* and in zebrafish have shown coordinated dynamic remodeling of synapses and dendritic arbor structure during tectal neuron development [18, 19]. In control neurons co-expressing tdTomato and PSD95-GFP, new PSD95-GFP postsynaptic specializations are added and stabilized within every 2 h of imaging (Fig. 8a; see also [19]). Consistent with the increased dendrite remodeling induced by netrin-1, *in vivo* imaging revealed that more PSD95-GFP-labeled postsynaptic specializations were added within the first observation interval in comparison to controls (0–2 h; Fig. 8a, b, Fig. 9a). Additionally, in netrin-treated tadpoles, relatively fewer postsynaptic specializations were stabilized 4 h following treatment when compared to controls (2- to 4-h interval; Figs. 8b, 9b). Treatment with UNC5H2-Ig did not significantly alter PSD95-GFP puncta addition or stabilization at any of the observation intervals although postsynaptic specializations tended to be less stable as more branches were eliminated after UNC5H2-Ig treatment (Figs. 8c, 9b). Surprisingly, even though dendrite remodeling occurred at the same rate as controls following anti-DCC treatment (Fig. 7 above), relatively more PSD95-GFP puncta were added during the first 2-h observation interval and fewer were stabilized between 2–4 h (Figs. 8d, 9a, b), similar to the effects of netrin-1.

To determine if PSD95-GFP puncta newly added in response to netrin-1 or anti-DCC treatment were more likely to be destabilized and eliminated, we then analyzed a subset of neurons to determine the fate of each individual puncta 4 h after treatment (arrows, Fig. 8). New puncta added from 0–2 h were significantly more likely to be eliminated at the 2- to 4-h interval following netrin-1 or anti-DCC treatment (control 11.67 ± 7.39 % *n* = 4; netrin 48.98 ± 12.36 % *n* = 4; anti-DCC 66.47 ± 12.33 % *n* = 4; Fig. 9c), indicating that active postsynaptic site remodeling accompanied dendrite branch remodeling. Even though manipulations in netrin levels and in DCC signaling significantly influenced postsynaptic specialization dynamics (increased addition followed by decreased stabilization), the density of PSD95-GFP puncta was not significantly different from controls at any of the observation time points in neurons from netrin-1-, anti-DCC-, or UNC5H2-Ig-treated tadpoles (i.e., at 0–4 h; control 131.2 ± 18.57 %, netrin 89.47 ± 6.823 %; UNC5H2-Ig 132.2 ± 13.98 %; anti-DCC 152.2 ± 36.10, *p* = 0.2537; one-way ANOVA, Dunnett’s multiple comparison test, not shown graphically).

### Manipulations in netrin signaling impact dendritic arbor directionality in multiple ways

**Fig. 10:**
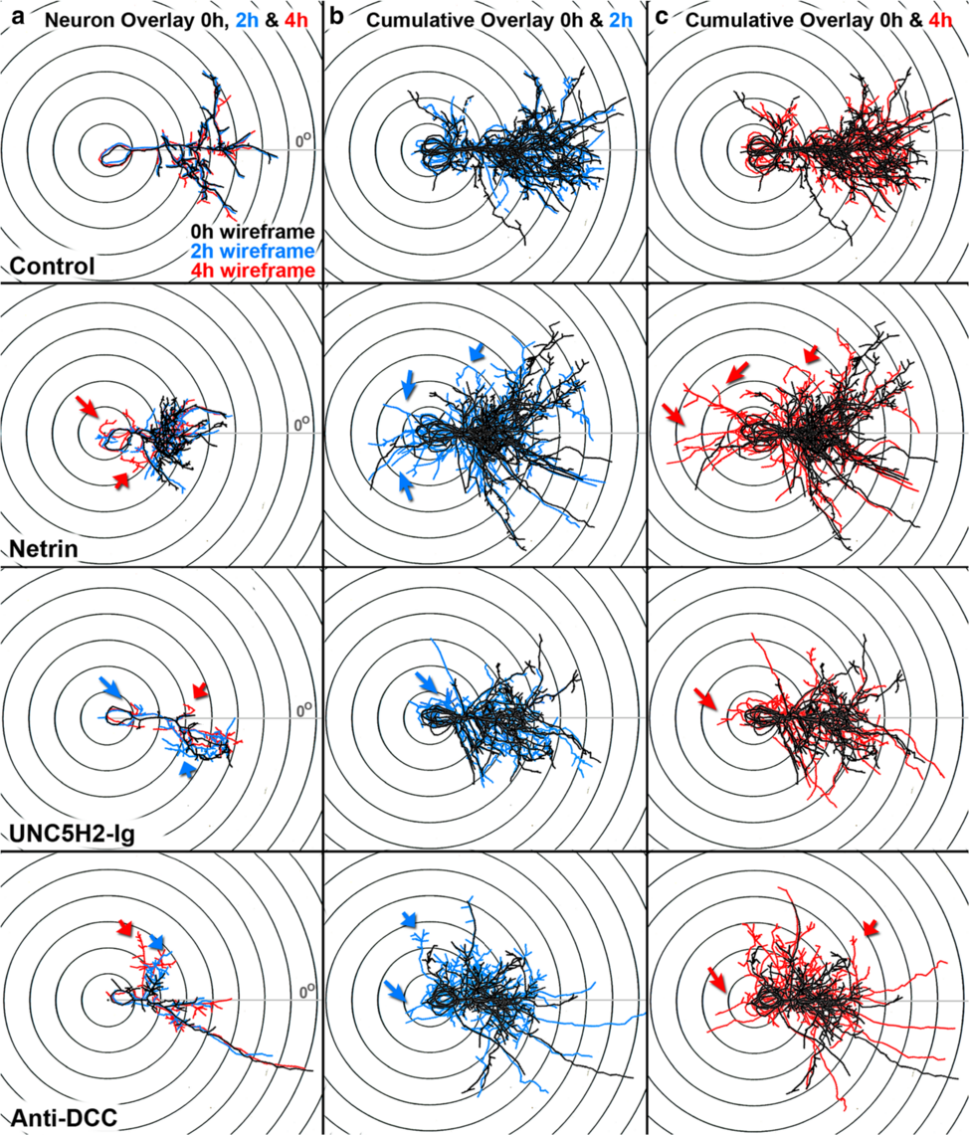
Overlays of sample neurons at 0, 2, and 4 h illustrate changes in dendritic arbor morphology in response to treatment and between imaging intervals. **a** Confocal stacks of individual neurons from control, netrin-1-, UNC5H2-Ig-, and anti-DCC-treated tadpoles were reconstructed with MetaMorph creating three-dimensional wireframes of each stack. Wireframes were color-coded based on imaging time point (*black*, 0 h; *blue*, 2 h; *red*, 4 h), overlapped, and aligned over Scholl concentric circles with the primary dendrite placed at a 0° angle (*X*-axis; *gray line*). Dynamic changes in dendritic morphology every 2 h over a 4-h imaging period are illustrated by the emergence of *blue* (2 h) or *red branches* (4 h) from under the *black wireframe* (0 h). **b, c** Cumulative wireframes from a subset of seven neurons per condition better illustrate the dynamic changes in growth between the 0- and 2-h imaging interval (**b**), and the 0- and 4-h imaging interval (**c**), for each treatment group. *Large arrows* point to sample ectopic branches newly extended at the time point indicated by the color of the arrow (*blue*, 2 h; *red*, 4 h). *Short arrows* point to already established branches that changed their directionality of growth at the time point indicated by the color of the arrow (*blue*, 2 h; *red*, 4 h)

**Fig. 11:**
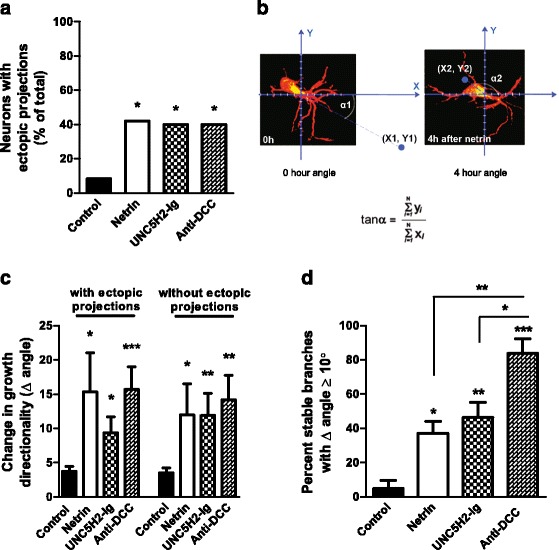
Perturbations in tectal netrin levels or signaling alter dendritic arbor directionality. **a** Proportion of neurons that developed ectopic basal projections within the 24-h period in each group. **b** Angle analysis performed on tectal neuron arbors sums all branch points to produce a net vector. The angle change was calculated from the tangents of arbors from 0 to 4 h. **c** The change in dendritic arbor directionality is shown as the difference in angle for neurons from 0 to 4 h and was measured both including (with) and excluding (without) ectopic projections. **d** Proportion of stable branches with net angle change. The percentage of stable branches that individually changed their angle by at least 10° was calculated for a subset of randomly selected neurons (*n* = 4). The individual branch tip vectors for each branch were compared from 0 to 4 h to calculate the angle change. Note that a larger proportion of stable branches altered their angle in neurons following anti-DCC treatment when compared to all other groups. Statistical significance was by Kruskal-Wallis Friedman with Dunn’s multiple comparison test. Significance when compared to control is *p < 0.05, **p < 0.01, ***p < 0.001. Error bars indicate SEM

Neurons in the optic tectum grow apical dendrites towards the tectal neuropil where they normally partner with RGC axons (Fig. 4a, b, d, and Fig. 6a). *In vivo* imaging showed that following netrin-1 treatment tectal neurons extended new ectopic basal projections, including a potential alternative primary dendrite (identified by the accumulation of PSD95-GFP, Fig. 4e, arrow) towards the ventricle midline while pruning or redirecting branches that normally grow towards the neuropil (Fig. 4e). Overlays of color-coded tracings (wireframes) of sample neurons imaged at 0, 2, and 4 h, as well as cumulative wireframes of a subset of neurons from each group, further illustrate the emergence of ectopic projections and dynamic changes in dendritic arbor growth in response to netrin-1, UNC5H2-Ig, or anti-DCC treatment (Fig. 10). The number of neurons that extended an alternative ectopic projection was significantly higher in netrin-treated tadpoles than in controls (control 8.33 %, netrin 42.11 %, *p* = 0.0131; Fisher’s exact test; Fig. 11a). Similar to netrin-1, either sequestering endogenous netrin with UNC5H2-Ig or altering DCC-mediated netrin signaling with anti-DCC resulted in a higher proportion of neurons that extended an ectopic projection away from the neuropil (control 8.33 %, anti-DCC 40.00 %, UNC5H2-Ig 40.00 %, *p* = 0.0370, Fisher’s exact test). To further evaluate changes in the orientation of the dendritic arbor, we calculated the vector angle for each neuron before and after treatment (Fig. 11b, see the “Methods” section). In the presence of exogenous netrin-1, neurons changed their vector angle within 4 h after treatment, a change that was significant whether alternative ectopic projections were included or excluded from the analysis (Fig. 11c). Neurons in anti-DCC- and in UNC5H2-Ig-treated tadpoles also remodeled and redirected their dendrites (Figs. 4f), effectively changing their vector and growth directionality within 4 h after treatment (Fig. 11c).

**Fig. 12:**
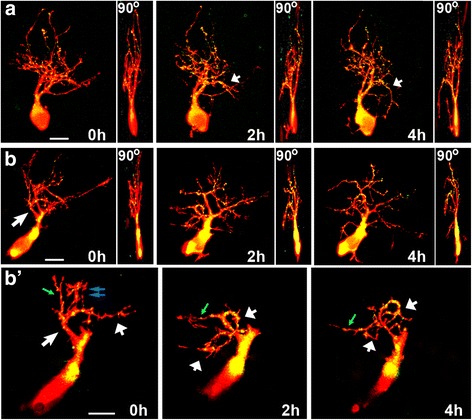
Individual branches change their orientation of growth in response to altered netrin levels. **a**, **b** The maximum projections of each confocal *z*-stack of two sample neurons at the 0-, 2-, and 4-h imaging time points, and the corresponding 90° view of each three-dimensional *z*-stack, illustrate the dynamic changes in growth and directionality of individual dendrites in response to acute netrin-1 treatment. The neuron in **a** corresponds to that shown in Fig. 5b. **b’** For the sample neuron in **b**, a single primary dendrite and its individual secondary branches of the same branch can be discerned in the higher magnification images by selecting and projecting only the *z*-planes from each confocal stack that include that branch. By isolating the individual dendrite from the rest of the dendritic arbor, one can better differentiate the change in the direction of growth of the primary dendrite (*short white arrows*) that took place while some of its secondary branches were pruned (*double blue arrows*) or changed their direction of growth (*green arrow*) and others were maintained. Scale bars: 20 μm

The effects of netrin-1, UNC5H2-Ig, and anti-DCC treatments indicate that even though all of the manipulations in netrin signaling significantly impact growth directionality in a relatively similar way, the mechanisms responsible for this remodeling may differ. Specifically, neurons in tadpoles treated with netrin-1 or UNC5H2-Ig showed dynamic dendrite branch remodeling that differed from those in tadpoles treated with anti-DCC, since anti-DCC did not affect new branch addition or branch stabilization rates (Fig. 7). *In vivo* imaging showed that some neurons seemed to grow or reorient their branch(es) in a direction opposite to the neuropil in response to treatment (Fig. 12, see also Fig. 4, inserts). To further differentiate whether the change in directionality resulted primarily from a reorientation of stable branches or from the addition of new branches with a different angle of growth, we analyzed a subset of neurons that showed a significant change in net vector angle by at least 10°. For this analysis, we determined the vector angle of each individual branch tip for all branches at both 0 and 4 h to determine the proportion of stable branches that changed their vector angle by more than 10° for every neuron in each group. Significantly more of the stable branches changed their vector angles in neurons of netrin-1- or UNC5H2-Ig-treated tadpoles relative to controls (Fig. 11d, ANOVA, Dunnett’s multiple comparison test). Moreover, significantly more of the stable branches changed their vector angle in neurons in anti-DCC-treated tadpoles than in any other treatment group (anti-DCC vs. netrin-1 *p* < 0.05, and *p* < 0.01 vs. UNC5H2-Ig, ANOVA, Tukey’s multiple comparison test), indicating that manipulations in netrin signaling influence arbor directionality by reorienting stable dendrites, while branch retraction and new branch extension also contribute to the reorganization of the dendritic arbor when threshold netrin levels and/or signaling are changed.

We performed a number of correlational analyses to further determine a potential relationship between the degree of neuronal maturation and a neuron’s response to altered netrin levels or DCC signaling. No significant correlation between a number of morphological parameters measured prior to treatment (total branch number or length, DCI value) and type of response (increased branch addition, decreased branch stabilization, vector angle change, ectopic dendrite growth) was found for neurons in either netrin-1- or UNC5H2-Ig-treated tadpoles at 4 and 24 h. This suggests that actively branching tectal neurons respond to altered midbrain netrin levels independently of their maturational state. Only younger, newly differentiated neurons with total branch number and DCI below the average at the initial observation time point were more likely to grow an ectopic dendrite following anti-DCC treatment (branch number *p* = 0.0031, DCI value *p* = 0.0311; chi-square), suggesting that in addition to maintaining stable dendrites, DCC-mediated netrin signaling can prevent the formation of ectopic dendrites during early phases of dendritic growth when most remodeling occurs [20].

### Sequestration of endogenous netrin-1 impacts visually guided behavior

**Fig. 13:**
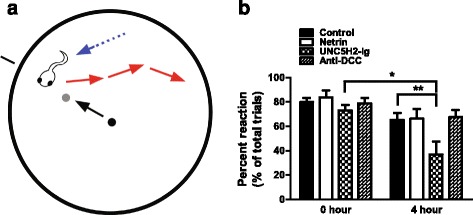
Sequestration of endogenous netrin-1 with UNC-5 ectodomain affects swimming behavior in a visually guided task. **a** Schematic of the visual avoidance task viewed from above. Stage 45 tadpoles swim in the 60-mm open field (*blue arrow* and *dotted line*) while the Matlab program projects an image on the monitor where the petri dish rests. The black line outside the field represents the vector the 0.3-mm dot (*small black circle*) will travel. Every 30 s, the 0.3-mm dot appears in the center and is directed towards the black line to intercept the tadpole (*black arrow*). The tadpole’s response to the advancing stimuli (*gray circle*) is video recorded and typically results in the tadpole changing its swimming velocity and/or direction (*red arrows*). **b** Reaction to the presentation of a moving visual stimulus for tadpoles before treatment (0 h) and 4 h after treatment with vehicle solution (control), netrin-1, anti-DCC, or UNC5H2-Ig is shown as the percent of trials in which tadpoles showed an avoidance response. Tadpoles injected with UNC5H2-Ig had decreased avoidance responses to the presentation of the stimulus 4 h post-injection. Two-way, repeated measures ANOVA with Bonferroni multiple comparison test; *p < 0.05, **p < 0.01. Error bars indicate SEM

Visual avoidance to moving light stimuli in *Xenopus* tadpoles is correlated with the maturation of visual responses in the optic tectum [21]. Deficits in visually guided behavior, in turn, have been correlated with abnormal visual system wiring [22, 23]. To test whether the netrin-induced changes in dendritic arbor morphology impact the functional organization of the retinotectal circuit, we used visually guided behavior as a functional assay. An avoidance behavior task [21] was adapted to probe specific visual responses of tadpoles at late stage 45 (Fig. 13a, see the “Methods” section). Tadpoles treated with netrin-1 or anti-DCC showed no changes in their ability to respond and avoid moving stimuli 4 h after treatment (Fig. 13b). In contrast to netrin-1 and anti-DCC, UNC5H2-Ig treatment resulted in abnormal visual avoidance behavior (Fig. 13b). Avoidance behavior of UNC5H2-Ig-treated tadpoles was significantly different from the behavior of the same tadpoles prior to treatment (0 h), as well as when compared to the behavior of tadpoles treated with either vehicle, netrin-1 or anti-DCC both at 0 and 4 h (avoidance at 0 h: vehicle 79.1 ± 3.2 %, UNC5H2-Ig 74.8 ± 4.5 %; avoidance at 4 h: vehicle 65.4 ± 2.91 %, UNC5H2-Ig 34.5 ± 7.2 %; *p* ≤ 0.005 two-way repeated measures ANOVA, *n* = 11–23 tadpoles per condition). The decreased ability of UNC5H2-Ig-treated tadpoles to respond to the moving stimuli was not due to alterations in their swimming capacity as the total swim time was not different for any of the treatment groups before or 4 h after treatment, whether tadpoles were presented with a moving dot (one-way ANOVA, not shown graphically, average swim time 57 ± 5.7 s out of 3-min total swim time/trial) or a video of a group of schooling tadpoles (data not shown). Consequently, sequestration of endogenous netrin-1 with UNC-5 ectodomain significantly influenced visually guided behavior in a rapid time scale, consistent with the significant dendrite remodeling effects and changes in tectal neuron morphology caused by the same treatment.

## Discussion

A growing number of molecules have been identified as factors that influence axon branching and synaptogenesis in the developing central nervous system. However, very few specific cues have been examined in real time to determine their influence on early dendritogenesis and dendrite arbor dynamics in the developing vertebrate brain. The observation that netrin-1, a molecule well known for its role in axon guidance, can also influence the steering and remodeling of central neuron dendrites in a manner that differs from its effects on presynaptic axons shows that netrin can modulate the structural plasticity of neurons in the vertebrate brain. In contrast to presynaptic RGC axons that stall and fail to further elaborate after blockade of DCC-mediated netrin signaling [11, 12], tectal neurons continued to branch but changed the orientation of their dendritic arbor, a response that suggests both direct and indirect effects.

Our studies show that the canonical netrin receptors, UNC-5 and DCC, are both expressed in the optic tectum. The localization of DCC to tectal neurons and dendritic processes and within the tectal neuropil supported a DCC-mediated mechanism by which netrin influences tectal neurons in addition to influencing RGC axons. Moreover, the expression of UNC-5 in subpopulations of tectal neurons that also express DCC suggested that both of these receptors could mediate responses to netrin-1. By examining dynamic changes in tectal neuron dendritic morphology and by directly correlating the changes of pre- and postsynaptic arbors in response to altered tectal netrin levels, our studies revealed differential effects of netrin-1 on tectal neurons and on RGC axons. In contrast to RGC axons that continued to arborize in response to netrin-1, tectal neurons pruned their dendrites away from the area co-occupied with the RGC axon within 4 h after netrin treatment, effectively remodeling their dendritic arbor (Fig. 4c). The effects of netrin on dendrites were more rapid and did not reflect the responses of RGC axon growth cones or of branching axons at the target [11, 12]. The rapid time course of netrin-1 action and its significant effects on dendrite remodeling therefore indicate that netrin can directly modulate postsynaptic neuronal morphology and connectivity in addition to influencing presynaptic RGC axons. Studies showing that altering the stability of presynaptic RGC axons upon decreased presynaptic neurotropic support only elicits time-delayed changes in the number of postsynaptic specializations in tectal neurons [19, 17] further support the idea that netrin-1 shapes postsynaptic neuronal connectivity directly.

Both midbrain injection of recombinant netrin-1 and sequestration of endogenous netrin by injection of UNC-5 ectodomain (UNC5H2-Ig) induced rapid tectal neuron dendritic remodeling and changed the orientation of dendritic growth. The observation that two treatments which increase and decrease bioavailable netrin had similar rather than opposite effects on developing neurons suggests that dendritic arbor remodeling or reorientation does not depend on the absolute concentration of netrin-1 but rather may be attributed to a change in the relative levels and/or distribution of the protein [24]. The findings that netrin mRNA is expressed near the ventricle wall in a pattern that differs from that of the secreted protein, and that targeted injection of netrin-1 into the ventricle and lateral side of the brain resulted in altered protein levels across the injected tectal hemisphere (Fig. 3), are consistent with a disruption of the relative levels of endogenous netrin-1 protein.

The observation that both netrin-1 and anti-DCC treatments had similar effects on dendrite directionality and on the formation and maintenance of post-synaptic specializations (adding more specializations in the 2 h immediately after injection and then subsequently removing 50–60 % of these same specializations by 4 h) suggests that netrin can shape tectal neuron morphology and synaptic connectivity by recruiting or binding to distinct receptors or multiple receptor complexes. It is possible that DCC contributes, at least in part, to both the morphological and synaptic effects of netrin on tectal neurons since neurons responded to acute changes in netrin-1 levels and to altered DCC signaling by effectively remodeling and reorienting their dendrites. Although quantitatively the dynamic changes differed among the treatment groups, the less robust but significant effects of anti-DCC on tectal neurons resulted in neurons with changes in the orientation of their stable dendrites and in the complexity of their dendritic arbor. The partial effect of the anti-DCC treatment therefore suggests that DCC may collaborate with other receptors to directly modulate tectal neuron differentiation. It is also possible that the effects of the anti-DCC treatment on tectal neurons are secondary to its effects on RGC axons [11, 12]. In addition to influencing axon arbors, DCC-mediated netrin signaling has been implicated in the synaptic differentiation of dendrites in multiple species [11, 25, 26]. Recent work indicating that DCC expression localizes on the tips of both dendrite and axon filopodia, is required for changes in actin filaments that precede filopodia remodeling, and can induce the enrichment of postsynaptic components in dendrites of cortical neurons in culture [25], are in agreement with our findings that DCC signaling can induce rapid changes in the recruitment of pre- and postsynaptic components *in vivo* in addition to influencing axon and dendrite branching. Studies demonstrating a role for DCC in sorting contralateral dendrites of hindbrain neurons in zebrafish larvae [27], and in modulating dendritic targeting in *Drosophila* sensory and motor neurons [13, 28, 14] and motor neuron dendritic growth in *C. elegans* [15], further support the idea that, as in other species, DCC-mediated netrin-1 signaling can influence tectal neuron dendritic differentiation directly.

The difference in responses of tectal neurons to altered netrin levels and to decreased DCC signaling and the co-expression patterns of DCC and UNC-5 indicate that DCC may signal independently or as a co-receptor with UNC-5. DCC and UNC-5 have previously been reported to form receptor complexes with one another to mediate repulsion during axon guidance [6, 29, 2] indicating the possibility that these receptors could similarly coordinate to affect dendritic arbor differentiation and maintenance. That UNC-5 signaling contributes to the effects we observed on dendrite orientation and branching is quite plausible, as sequestering endogenous netrin from all potential receptors with UNC5H2-Ig had more striking effects than treatment with anti-DCC alone. UNC-5 has been shown to induce neurite outgrowth in neuroblastoma cells in a netrin-1-dependent manner [30] and to modulate synaptic differentiation in motor neuron dendrites in *C. elegans* [26]. A number of studies have implicated UNC-5 receptor-mediated netrin signaling not only in the differentiation but also in the survival of neurons [31, 1, 32]. Observations that a larger proportion of tectal neurons underwent cell death between 8 and 24 h after UNC5H2-Ig treatment alone (UNC5H2-Ig 40 % vs. netrin + UNC5H2-Ig 0 %, *p* = 0.05 vs. control 19.2 %, *p* = 0.272; netrin + UNC5H2-Ig vs. control, *p* = 0.293; Fisher’s exact test) suggest that cell death could be a consequence of interfering with endogenous netrin-1 signaling in the *Xenopus* optic tectum and support the contribution of UNC-5 receptor signaling in the modulation of tectal neuron differentiation.

The responses of tectal neurons to acute manipulations that altered netrin levels may reflect the interplay of the influence of netrin-1 on developing tectal neurons and on RGC axons. RGC axons and tectal neurons seem to respond differently to the same manipulations that alter netrin levels or DCC signaling, in a way that can create a potential disconnect among pre- and postsynaptic neurons. Tectal neurons prune and remodel their dendrites when endogenous netrin levels are decreased while RGC axons fail to branch and differentiate in the absence of DCC signaling [11, 12]. The functional consequence of such a potential disconnect was demonstrated by our behavioral studies. UNC5H2-Ig treatment significantly influenced the tadpoles’ visual responses to a moving stimulus shortly after treatment, indicating that interfering with endogenous netrin signaling impacts functional connectivity. Studies demonstrating functional deficits to visual stimuli have mostly used chronic manipulations that significantly disrupt synaptic transmission and retinotectal circuit formation [21, 22], highlighting the rapid effects of altering netrin signaling. The observation that only sequestration of endogenous netrin-1 (that had the most significant effects on the morphology and growth of the dendritic arbor) influenced visually guided behavior but not treatment with netrin-1, however, suggests that the rapid postsynaptic specialization remodeling that occurred in response to acute netrin treatment may serve to maintain circuit connectivity by compensating for the dendrite remodeling effects of netrin-1. The time course of presynaptic axon responses to netrin-1, where RGC axons rapidly increase their presynaptic site density and dynamic branch behavior 4 h after netrin-1 treatment, ultimately increasing their branch number and size of the arbor [11], supports the idea that dynamic pre-and postsynaptic remodeling maintains retinotectal connectivity as tectal neurons reorient their dendritic arbors in the presence of excess netrin-1.

## Conclusions

How does netrin shape dendritic architecture in the *Xenopus* brain? Netrin-1 mRNA is expressed in the periventricular area of the midbrain, and netrin protein can be localized in both the cell body area and neuropil, supporting the possibility that secreted netrin diffuses away from the midline source [24] forming a ventro-dorsal and medial-lateral gradient which may be used by tectal dendrites to navigate. The observation that a significant portion of tectal neurons rapidly remodel and reorient their dendrites (by pruning their apically oriented dendrites and extending basal processes towards the midline) in response to acute alterations in endogenous netrin levels supports the idea that the change in directionality of dendrite growth may be a direct consequence of disturbing an endogenous netrin-1 gradient, similar to what has been shown for the early growth of cortical neuron dendrites in response to semaphorin 3A [33]. Dendrite remodeling and reorientation of arbor growth may instead be a response by the already polarized neurons to the change in local levels of netrin, partially reverting them to non-polarized growth. The presence of a shallow gradient of endogenous netrin immunoreactivity in the neuropil (Fig. 3c), the rapid diffusion of injected proteins across the tectal hemisphere, and the observation that the two treatments which increase and decrease bioavailable netrin had similar effects on developing neurons further suggest that dendritic arbor remodeling or reorientation does not depend on the absolute concentration or level of netrin-1 but rather may be attributed to altering threshold netrin signaling [34, 35] or to disturbing an endogenous netrin-1 gradient [24]. An intriguing possibility is that coordinated signaling of DCC and UNC-5 receptors, in a manner similar to their collaboration in guiding axonal processes [4, 5], allows these receptors to sense changes in relative netrin-1 levels in the developing midbrain and repel the dendrites of tectal neurons away from their birthplace along the ventricle to guide them towards their axonal targets in the neuropil. It is also possible that some of the effects of netrin-1 may be attributable to ligand-mediated downregulation of receptor function [36, 12] since treatment with function-blocking antibodies to DCC altered dendritic arbor orientation, similar to netrin-1. Secreted netrin may also be captured by receptors and/or extracellular matrix molecules which can then shape the spatial distribution of netrin protein within the tectum, similar to the function of DCC orthologues in *Drosophila* [37] and to collagen that can provide a signaling gradient for axon guidance cues in the vertebrate visual system [38]. Most interesting about these possibilities is that the differential distribution of netrin across the tectal cell body layer and within the neuropil could serve to coordinate both the postsynaptic dendrites and presynaptic axons. Axons expressing DCC would be attracted to the areas of increased netrin in the tectum while dendrites co-expressing both DCC and UNC-5 would be directed away from the midline and towards the neuropil. Differential signaling mechanisms by which vertebrate central neurons change their response to molecular signals, alone or in combination, to actively orient and maintain their dendrites are intriguing possibilities that remain open to further investigation.

## Methods

### Animals

*Xenopus laevis* tadpoles were obtained by *in vitro* fertilization of oocytes from adult females primed with human chorionic gonadotropin and raised in rearing solution [60 mM NaCl, 0.67 mM KCl, 0.34 mM Ca(NO3)2, 0.83 mM MgSO4, 10 mM HEPES, pH 7.4, and 40 mg/l gentamycin] plus 0.001 % phenylthiocarbamide to prevent melanocyte pigmentation. Tadpoles were anesthetized during experimental manipulations with 0.05 % tricaine methanesulfonate (Finquel; Argent Laboratories, Redmond, WA, USA). Staging was performed according to Nieuwkoop and Faber [39]. Animal procedures were approved by the Institutional Animal Care and Use Committee of the University of California, Irvine (Animal Welfare Assurance Number A3416-01).

### *In situ* hybridization

A *Xenopus*-specific netrin-1 cDNA was a generous gift of Dr. Christine Holt [40, 10]. For *in situ* hybridization, stage 45 tadpoles were anesthetized and fixed for 2 h in 4 % paraformaldehyde in phosphate buffer (PB), pH 7.5. Coronal cryostat sections (40 μm) were hybridized with DIG-11-UTP-labeled antisense and sense RNA probes as described previously [41]. After hybridization, sections were washed, incubated overnight with an alkaline phosphatase-coupled anti-DIG antibody, and developed with a BCIP/NBT Color Development Substrate (Promega, Madison, WI, USA). Endogenous netrin-1, UNC-5, and DCC mRNA expression within the tectum were independently confirmed by quantitative RT-PCR (not shown).

### Immunohistochemistry

Stage 45 tadpoles were euthanized with tricaine methanesulfonate and fixed in 4 % paraformaldehyde in PB, pH 7.5, for 2 h. For coronal sections, tadpoles were cryoprotected in 30 % sucrose overnight and embedded in OCT compound (Sakura Finetek, Torrance, CA, USA), and 40-μm cryostat sections were obtained. For horizontal sections, brains were then dissected out, embedded in 2 % agarose, and sectioned into 50-μm slices using a vibratome. Coronal and horizontal sections at the level of the optic tectum were incubated with the following primary antibodies without antigen retrieval step [11]: mouse monoclonal antihuman presynaptic protein SNAP-25 (1:500 dilution; Enzo Life Science, Farmingdale, NY, USA), mouse monoclonal antibody against the extracellular domain of human DCC (1:100 dilution; anti-DCC, Genetex Clone AF5, Irvine, CA, USA), mouse monoclonal antibody against the intracellular domain of human DCC (1:1500 dilution; BD Biosciences Pharmingen, San Jose, CA, USA), chicken polyclonal antibody against human netrin-1 (1:3500, Novus Biologicals, Littleton, CO, USA), and rabbit polyclonal anti-mouse UNC-5H3 antibody (1:14,000 dilution; generous gift of Dr. Antony Pawson). Primary antibodies were visualized using donkey anti-mouse and anti-rabbit, Alexa 488 and 568, or goat anti-chicken Alexa 488 secondary antibodies (1:500 dilution; Life Technologies, Grand Island, NY, USA). The specificity of the antibodies to recognize *Xenopus* UNC-5 and DCC was tested by Western blot analysis: a band of ∼ 180 kDa was detected by the anti-DCC antibodies in stage 45 tectum, and a band of ~145 kDa was detected by the anti-UNC5H3 antibody in stage 45 tectum, consistent with the predicted molecular weight of *Xenopus* DCC and UNC-5, respectively (not shown). In some experiments, RGC axons were anterogradely labeled by iontophoresis of rhodamine-dextran amine (10 % *w*/*v*; 3000 MW lysine fixable; Molecular Probes, Eugene, OR, USA) into the right eye of anesthetized, stage 42 tadpoles prior to fixation and immunostaining.

### Single cell transfection, tadpole treatment, and *in vivo* imaging

Co-transfection of tectal neurons and RGCs was performed by pressure injection of tdTomato and enhanced green fluorescent protein (EGFP; Clontech, Palo Alto, CA, USA) expression plasmids mixed with DOTAP liposomal transfection reagent (10 nl solution of 1 μg/μl plasmid; Roche Diagnostics, Indianapolis, IN, USA) into the brain primordia and contralateral eye, respectively, of anesthetized stage 20–22 tadpoles. In other experiments, to visualize dendritic morphology and postsynaptic specializations simultaneously in individual tectal neurons, brain progenitor cells were co-transfected with tdTomato and PSD95-GFP expression plasmids [19]. Tadpoles were reared until stage 45, when tadpoles with individually labeled neurons with at least seven dendritic branches were selected for imaging. Following the first imaging session (0 h), 30 nl of vehicle solution (0.1 % BSA, 50 % Niu Twitty), recombinant chicken netrin-1 (300 ng/μl), rat UNC5H2 Fc chimera (UNC5H2-Ig; 300 ng/μl), function--blocking antibody to DCC (50 ng; GeneTex (Irvine, CA, USA), anti-DCC, AF5), or recombinant human IgG1 Fc (R&D Systems Inc., Minneapolis, MN, USA) was pressure injected both medially and laterally into the ventricle and the subpial space overlying the optic tectum. Co-injection of recombinant netrin and UNC5H2-Ig (netrin + UNC5H2-Ig; 17 ng/30 ng) was used to control for the netrin and UNC5H2-Ig treatments alone. The concentration of UNC5H2-Ig and of recombinant netrin-1 was calculated to provide an excess of netrin that would bind to the dimerized UNC5H2-Ig chimera, thereby preventing both from binding endogenous ligand or receptors and from potentially masking endogenous netrin-1 gradients. For all measures before and after treatment, neurons from tadpoles co-treated with netrin + UNC5H2-Ig were similar to controls. After injection, tadpoles were imaged every 2 h for 4 h and then again at 24 h. Only neurons that were accessible to imaging and intact 8 h after initial imaging were included in the analysis. Images were acquired using LSM 5 Pascal confocal microscope with a × 63/0.95 water immersion objective. Optical sections were collected at 1.2-μm intervals. Diffusion of injected proteins was confirmed by immunohistochemistry on groups of non-imaged animals fixed immediately after treatment. Diffusion of recombinant netrin was analyzed using MetaMorph. A quantitative measure of the relative intensity of the immunofluorescent signals was obtained from confocal images acquired with identical laser capture settings from brains of untreated, vehicle-injected, and netrin-1-injected tadpoles. The average pixel intensity values (gray level, 255 maximum) in 20-pixel-wide line scans along the medial to lateral axis of the tectum (from the ventricle to the lateral-most side of the tectum, excluding pia and skin) were measured with MetaMorph; pixel intensity values were averaged for every 5 μm and normalized to those at the highest intensity value for each group.

### Data analysis

In brief, digital three-dimensional reconstructions of EGFP-labeled RGC axons or tdTomato and PSD95-GFP double-labeled tectal neurons were analyzed as before [19, 11] with the aid of the MetaMorph software (Molecular Devices, Sunnyvale, CA, USA) without any post-acquisition manipulation or thresholding. Processes of more than 5 μm in length were considered branches. For RGC axons, we measured total axon branch number and length. For tectal neurons, several morphological parameters were measured: total dendrite number and total dendritic arbor length and addition and stability of individual branches. To characterize the distribution of PSD95-GFP puncta to particular regions in tectal neuron dendritic arbors, pixel-by-pixel overlaps of individual optical sections obtained at the two wavelengths were analyzed. Addition and stability of PSD95-GFP-labeled puncta and postsynaptic specialization density (the number of PSD95-GFP puncta per 10 μm) were determined. Changes from each observation time point relative to 0 h, as well as from a given time point relative to the previous time point, were calculated and are expressed as percentages. A change in directionality of dendritic growth was calculated using two-dimensional digital arbor reconstructions. For each projection, a straight line was drawn connecting the point where the primary dendrite emerges from the cell body and the first primary dendrite bifurcation. This line was selected as the *X*-axis, with its center positioned in the middle of the cell body. The directionality vector was determined for each arbor by summation of *X*- and *Y*-coordinates of all branch tips. The directionality vector angle was determined in relation to the *X*-axis, and the difference in vector angles between 0- and 4-h projections was calculated for each neuron.

A total of 10–26 tectal neurons were analyzed per condition (control *n* = 26, netrin *n* = 19, UNC5H2-Ig *n* = 15, UNC5H2-Ig + netrin *n* = 10, anti-DCC *n* = 15) unless otherwise noted in the text, with one tectal neuron analyzed per tadpole. Dendritic arbors in tadpoles injected with control, recombinant human IgG Fc exhibited branch and PSD95-GFP cluster dynamics comparable to those of vehicle-treated tadpoles and were therefore grouped as controls. For all analyzed measures, neurons in netrin-1-, UNC5H2-Ig-, netrin + UNC5H2-Ig-, or anti-DCC-treated tadpoles did not differ significantly from controls prior to treatment (branch number at 0 h: control 15.35 ± 0.92, netrin 17.4 ± 1.9, UNC5H2-Ig 13.71 ± 1.0, anti-DCC 13.06 ± 0.96, netrin + UNC5H2-Ig 13.72 ± 1.91; dendritic arbor length at 0 h: control 327.10 ± 93.93 μm, netrin 341.10 ± 147.0 μm, UNC5H2-Ig 245.00 ± 86.37 μm, anti-DCC 280.20 ± 92.36 μm, netrin + UNC5H2-Ig 333.46 ± 41.70 μm; dendritic complexity index at 0 h: control 2.29 ± 0.05, netrin 2.33 ± 0.08, UNC5H2-Ig 2.32 ± 0.07, anti-DCC 2.20 ± 0.06; PSD95-GFP puncta number at 0 h: control 32.63 ± 4.27, netrin 33.58 ± 4.10, UNC5H2-Ig 21.5 ± 2.53, anti-DCC 25.7 ± 4.90; netrin + UNC5H2-Ig, 38.0 ± 7.19). Two-way ANOVA with Bonferroni multiple comparison or one-way ANOVA with Tukey’s multiple comparison tests were used for the statistical analysis of data. Results were considered significant in comparison to control as follows: *p ≤ 0.05, **p ≤ 0.005, ***p ≤ 0.001, unless otherwise indicated on the graph with bars marking additional significant comparisons.

### Visual avoidance task

Stage 45 tadpoles were placed in a 60 mm × 20 mm clear plastic petri dish, with darkened walls, filled to a depth of 1 cm with modified rearing solution at room temperature. The dish was placed on a CRT monitor screen and a solid, opaque box was placed over the monitor to eliminate outside light. A camera was affixed to the opening at the top of the box for video recording. Visual stimuli was produced by a custom-written Matlab program (MathWorks, Natick, MA, USA) generously donated by Dr. Carlos Aizenman, Brown University. A black circle with radius 0.3 mm was projected in the center of a circle on a white background. This size was found to produce optimal responses to the stimulus as shown in [21]. The circle was then manually directed to collide with the path of the swimming tadpole every 30 s for six trials. The tadpole’s responses to the circle, when the dot approached the tadpole and when the dot returned to the dish center, were analyzed blind to treatment with frame-by-frame replay of recorded responses. Tadpoles were observed to both freeze and swim away by altering their direction, speed, or both when presented with stimuli. These responses were counted as visual reactions to the stimuli. Failure to move away from the circle or a lack of freezing behavior prior to when the circle encountered the tadpole was considered a failure to respond. Experiments were performed during the 12-h light cycle. Treatments were identical to those of *in vivo* imaging studies with the exception that tadpoles were injected in the ventricle and laterally in the subpial space overlying both tectal hemispheres. Only tadpoles that responded to at least 50 % of the visual stimuli at 0 h were included in the analysis. The behavior of a total of 11–23 tadpoles was analyzed per condition (control *n* = 23 (9 vehicle-treated, 14 non-immune IgG-treated), netrin *n* = 11, UNC5H2-Ig *n* = 13, anti-DCC *n* = 12). Repeated measures, two-way ANOVA with Bonferroni multiple comparison test, or one-way ANOVA with Tukey’s multiple comparison tests were used for the statistical analysis of the data. Results were considered significant as follows: *p ≤ 0.05, **p ≤ 0.005, ***p ≤ 0.001.

## Abbreviations

ANOVA: analysis of variance
DCC: deleted in colorectal cancer
DCI: dendritic complexity index
EGFP: enhanced green fluorescent protein
if: infundibulum
np: tectal neuropil
RGC: retinal ganglion cell
UNC-5: uncoordinated-5
vm: ventral midline

## References

[1] Lai Wing Sun K, Correia JP, Kennedy TE (2011). Netrins: versatile extracellular cues with diverse functions. Development.

[2] Finci LI, Kruger N, Sun X, Zhang J, Chegkazi M, Wu Y (2014). The crystal structure of netrin-1 in complex with DCC reveals the bifunctionality of netrin-1 as a guidance cue. Neuron.

[3] Xu K, Wu Z, Renier N, Antipenko A, Tzvetkova-Robev D, Xu Y (2014). Neural migration. Structures of netrin-1 bound to two receptors provide insight into its axon guidance mechanism. Science.

[4] Chan SS, Zheng H, Su MW, Wilk R, Killeen MT, Hedgecock EM (1996). UNC-40, a C. elegans homolog of DCC (deleted in colorectal cancer), is required in motile cells responding to UNC-6 netrin cues. Cell.

[5] Keino-Masu K, Masu M, Hinck L, Leonardo ED, Chan SS, Culotti JG (1996). Deleted in colorectal cancer (DCC) encodes a netrin receptor. Cell.

[6] Hong K, Hinck L, Nishiyama M, Poo MM, Tessier-Lavigne M, Stein E (1999). A ligand-gated association between cytoplasmic domains of UNC5 and DCC family receptors converts netrin-induced growth cone attraction to repulsion. Cell.

[7] Keleman K, Dickson BJ (2001). Short- and long-range repulsion by the Drosophila Unc5 netrin receptor. Neuron.

[8] Merz DC, Zheng H, Killeen MT, Krizus A, Culotti JG (2001). Multiple signaling mechanisms of the UNC-6/netrin receptors UNC-5 and UNC-40/DCC in vivo. Genetics.

[9] Deiner MS, Sretavan DW (1999). Altered midline axon pathways and ectopic neurons in the developing hypothalamus of netrin-1- and DCC-deficient mice. J Neurosci.

[10] Shewan D, Dwivedy A, Anderson R, Holt CE (2002). Age-related changes underlie switch in netrin-1 responsiveness as growth cones advance along visual pathway. Nat Neurosci.

[11] Manitt C, Nikolakopoulou AM, Almario DR, Nguyen SA, Cohen-Cory S (2009). Netrin participates in the development of retinotectal synaptic connectivity by modulating axon arborization and synapse formation in the developing brain. J Neurosci.

[12] Shirkey NJ, Manitt C, Zuniga L, Cohen-Cory S (2012). Dynamic responses of Xenopus retinal ganglion cell axon growth cones to netrin-1 as they innervate their in vivo target. Dev Neurobiol.

[13] Furrer MP, Kim S, Wolf B, Chiba A (2003). Robo and frazzled/DCC mediate dendritic guidance at the CNS midline. Nat Neurosci.

[14] Matthews BJ, Grueber WB (2011). Dscam1-mediated self-avoidance counters netrin-dependent targeting of dendrites in Drosophila. Curr Biol.

[15] Teichmann HM, Shen K (2011). UNC-6 and UNC-40 promote dendritic growth through PAR-4 in Caenorhabditis elegans neurons. Nat Neurosci.

[16] Low K, Culbertson M, Bradke F, Tessier-Lavigne M, Tuszynski MH (2008). Netrin-1 is a novel myelin-associated inhibitor to axon growth. J Neurosci.

[17] Marshak S, Nikolakopoulou AM, Dirks R, Martens GJ, Cohen-Cory S (2007). Cell-autonomous TrkB signaling in presynaptic retinal ganglion cells mediates axon arbor growth and synapse maturation during the establishment of retinotectal synaptic connectivity. J Neurosci.

[18] Niell CM, Meyer MP, Smith SJ (2004). In vivo imaging of synapse formation on a growing dendritic arbor. Nat Neurosci.

[19] Sanchez AL, Matthews BJ, Meynard MM, Hu B, Javed S, Cohen CS (2006). BDNF increases synapse density in dendrites of developing tectal neurons in vivo. Development.

[20] Wu GY, Zou DJ, Rajan I, Cline H (1999). Dendritic dynamics in vivo change during neuronal maturation. J Neurosci.

[21] Dong W, Lee RH, Xu H, Yang S, Pratt KG, Cao V (2009). Visual avoidance in Xenopus tadpoles is correlated with the maturation of visual responses in the optic tectum. J Neurophysiol.

[22] Lee RH, Mills EA, Schwartz N, Bell MR, Deeg KE, Ruthazer ES (2010). Neurodevelopmental effects of chronic exposure to elevated levels of pro-inflammatory cytokines in a developing visual system. Neural Dev.

[23] Spawn A, Aizenman CD (2012). Abnormal visual processing and increased seizure susceptibility result from developmental exposure to the biocide methylisothiazolinone. Neuroscience.

[24] Kennedy TE, Wang H, Marshall W, Tessier-Lavigne M (2006). Axon guidance by diffusible chemoattractants: a gradient of netrin protein in the developing spinal cord. J Neurosci.

[25] Goldman JS, Ashour MA, Magdesian MH, Tritsch NX, Harris SN, Christofi N (2013). Netrin-1 promotes excitatory synaptogenesis between cortical neurons by initiating synapse assembly. J Neurosci.

[26] Poon VY, Klassen MP, Shen K (2008). UNC-6/netrin and its receptor UNC-5 locally exclude presynaptic components from dendrites. Nature.

[27] Suli A, Mortimer N, Shepherd I, Chien CB (2006). Netrin/DCC signaling controls contralateral dendrites of octavolateralis efferent neurons. J Neurosci.

[28] Mauss A, Tripodi M, Evers JF, Landgraf M (2009). Midline signalling systems direct the formation of a neural map by dendritic targeting in the Drosophila motor system. PLoS Biol.

[29] Norris AD, Lundquist EA (2011). UNC-6/netrin and its receptors UNC-5 and UNC-40/DCC modulate growth cone protrusion in vivo in C. elegans. Development.

[30] Picard M, Petrie RJ, Antoine-Bertrand J, Saint-Cyr-Proulx E, Villemure JF, Lamarche-Vane N (2009). Spatial and temporal activation of the small GTPases RhoA and Rac1 by the netrin-1 receptor UNC5a during neurite outgrowth. Cell Signal.

[31] Llambi F, Causeret F, Bloch-Gallego E, Mehlen P (2001). Netrin-1 acts as a survival factor via its receptors UNC5H and DCC. EMBO J.

[32] Tang X, Jang SW, Okada M, Chan CB, Feng Y, Liu Y (2008). Netrin-1 mediates neuronal survival through PIKE-L interaction with the dependence receptor UNC5B. Nat Cell Biol.

[33] Polleux F, Morrow T, Ghosh A (2000). Semaphorin 3A is a chemoattractant for cortical apical dendrites. Nature.

[34] Ming GL, Wong ST, Henley J, Yuan XB, Song HJ, Spitzer NC (2002). Adaptation in the chemotactic guidance of nerve growth cones. Nature.

[35] Piper M, Salih S, Weinl C, Holt CE, Harris WA (2005). Endocytosis-dependent desensitization and protein synthesis-dependent resensitization in retinal growth cone adaptation. Nat Neurosci.

[36] Kim TH, Lee HK, Seo IA, Bae HR, Suh DJ, Wu J (2005). Netrin induces down-regulation of its receptor, deleted in colorectal cancer, through the ubiquitin-proteasome pathway in the embryonic cortical neuron. J Neurochem.

[37] Hiramoto M, Hiromi Y, Giniger E, Hotta Y (2000). The Drosophila netrin receptor frazzled guides axons by controlling netrin distribution. Nature.

[38] Xiao T, Staub W, Robles E, Gosse NJ, Cole GJ, Baier H (2011). Assembly of lamina-specific neuronal connections by slit bound to type IV collagen. Cell.

[39] Nieuwkoop PD, Faber J (1956). Normal table of Xenopus laevis.

[40] Anderson RB, Holt CE (2002). Expression of UNC-5 in the developing Xenopus visual system. Mech Dev.

[41] Cohen-Cory S, Fraser SE (1994). BDNF in the development of the visual system of Xenopus. Neuron.

[42] Furne C, Rama N, Corset V, Chedotal A, Mehlen P (2008). Netrin-1 is a survival factor during commissural neuron avigation. Proc Natl Acad Sci U S A.

[43] Dillon AK, Fujita SC, Matise MP, Jarjour AA, Kennedy TE, Kollmus H (2005). Molecular control of spinal accessory motor neuron/axon development in the mouse spinal cord. J Neurosci.

[44] Dillon AK, Jevince AR, Hinck L, Ackerman SL, Lu X, Tessier-Lavigne M (2007). UNC5C is required for spinal accessory motor neuron development. Mol Cell Neurosci.

[45] Kennedy TE (2000). Cellular mechanisms of netrin function: long-range and short-range actions. Biochem Cell Biol.

